# Uncovering information on expression of natural antisense transcripts in Affymetrix MOE430 datasets

**DOI:** 10.1186/1471-2164-8-200

**Published:** 2007-06-28

**Authors:** Sebastian Oeder, Joerg Mages, Paul Flicek, Roland Lang

**Affiliations:** 1 Institute of Medical Microbiology, Immunology and Hygiene Technical University Munich Trogerstr, 30 81675, Munich, Germany; 2 European Bioinformatics Institute Wellcome Trust Genome Campus Hinxton, Cambridge, CB10 1SD, UK

## Abstract

**Background:**

The function and significance of the widespread expression of natural antisense transcripts (NATs) is largely unknown. The ability to quantitatively assess changes in NAT expression for many different transcripts in multiple samples would facilitate our understanding of this relatively new class of RNA molecules.

**Results:**

Here, we demonstrate that standard expression analysis Affymetrix MOE430 and HG-U133 GeneChips contain hundreds of probe sets that detect NATs. Probe sets carrying a "Negative Strand Matching Probes" annotation in NetAffx were validated using Ensembl by manual and automated approaches. More than 50 % of the 1,113 probe sets with "Negative Strand Matching Probes" on the MOE430 2.0 GeneChip were confirmed as detecting NATs. Expression of selected antisense transcripts as indicated by Affymetrix data was confirmed using strand-specific RT-PCR. Thus, Affymetrix datasets can be mined to reveal information about the regulated expression of a considerable number of NATs. In a correlation analysis of 179 sense-antisense (SAS) probe set pairs using publicly available data from 1637 MOE430 2.0 GeneChips a significant number of SAS transcript pairs were found to be positively correlated.

**Conclusion:**

Standard expression analysis Affymetrix GeneChips can be used to measure many different NATs. The large amount of samples deposited in microarray databases represents a valuable resource for a quantitative analysis of NAT expression and regulation in different cells, tissues and biological conditions.

## Background

Natural antisense transcripts (NATs) are RNA molecules harboring sequences complementary to other transcripts. Expression of endogenous NATs was first observed in viruses [1] and prokaryotes [2], followed by its detection also in eukaryotes [3]. The number of sense-antisense (SAS) transcripts slowly grew during the 1990's, as more examples were found in the study of the regulation of expression of individual genes. In some of these cases, a regulatory function for antisense RNA in controlling the mRNA or protein levels of the respective sense gene product was described (for reviews see ref. [4,5]). The availability of large scale cDNA sequencing then led to the in silico discovery of significant numbers of SAS transcript pairs in different species, including yeast, plants and mammals [6-14]. Most recently, systematic genome-wide detection of transcription by strand-specific tiling oligonucleotide arrays or sequencing (CAGE) substantiated the notion that expression of antisense transcripts is a widespread phenomenon, accounting for up to 50 % of all transcripts [15-17]. To date, only few studies have analyzed quantitative changes in the expression of larger numbers of NATs under different experimental conditions. The function(s) of NATs and the regulation of their expression in relation to the corrresponding sense transcripts are largely unknown [4,18]. One possible function of NATs is decreasing sense transcript abundance, e.g. by RNAi-mediated degradation [19]. In this case, the levels of sense and antisense transcript would be expected to be negatively correlated. The effects of NATs are not necessarily mediated by RNA-RNA hybridization but can also be brought about by RNA-protein interactions [20,21].

Oligonucleotide microarrays such as Affymetrix GeneChips detect labeled cRNA in a strand-specific manner. Standard labeling methods based on incorporation of a T7 binding site with an oligo-dT primer during first strand cDNA synthesis not only amplify selectively polyA mRNA, but also conserve strand-specificity of the resulting cRNA. This technology therefore in principle allows the measurement of transcripts in an orientation-specific manner. Tiling arrays have indeed been used for a detailed experimental investigation of transcriptional activity on human chromosomes [15,22,23]. These studies have greatly advanced our understanding of the extent of genome-wide transcriptional activity and the abundance of NATs and other non-coding RNA transcripts. However, the requirement of multiple arrays to perform a genome-wide tiling array analysis of a single RNA sample makes larger experiments prohibitively expensive, and therefore has to date precluded a comprehensive investigation of NATs expression and regulation in many different experimental conditions. In contrast, expression analysis GeneChips such as MOE430 for murine and U133 for human RNA detect and measure a comprehensive set of the majority of protein coding mRNAs on one single microarray, making global mRNA expression profiling experiments relatively affordable. Their extensive use in functional genomics studies has resulted in a wealth of data sets deposited in public data repositories. In this paper, we point to the presence of a considerable number of probe sets detecting NATs on Affymetrix GeneChip expression analysis arrays. Probe sets potentially detecting NAT were validated *in silico*, and microarray results showing expression of NATs were confirmed by strand-specific RT-PCR. The large number of Affymetrix datasets in public domain repositories like Gene Expression Omnibus (GEO) can therefore be mined for the expression and regulation of hundreds of NATs.

## Results and Discussion

### Affymetrix GeneChip probe sets with "Negative Strand Matching Probes": in silico validation

**Figure 1:**
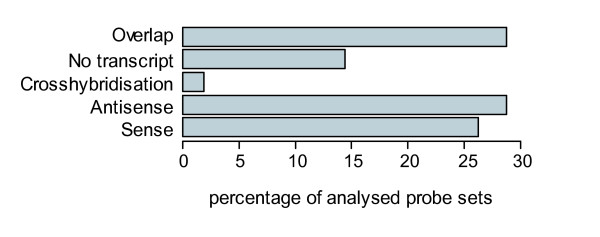
**In silico validation of potential NAT-detecting Affymetrix probe sets**. Using an automated classification algorithm, 1,113 probe sets annotated by Affymetrix as *Negative Strand Match Probes *were assigned to the displayed categories based on the orientation of the probe set sequence ("sense", "antisense", "overlap") found. Also depicted is the frequency of cross-hybridizing probe sets and those without match to an annotated transcript.

The annotation tables of Affymetrix GeneChips contain an entry termed "Negative Strand Matching Probes" [24], suggesting that the respective probe sets may detect not the expression of the sense transcript indicated by the gene symbol for the probe set but instead a NAT. Of the 45 K probe sets on the murine GeneChip MOE430 2.0 1113 carry this annotation for all 11 probes of the probe set, while the same applies to 3141 probe sets on the human HG U133 Plus 2.0. To determine whether this annotation could indeed be used to filter for probe sets detecting NAT expression, we developed an automatic classification algorithm based on the probe set mapping to the Ensembl v36 genome release [25]. Probe sets that did not map to a transcript at all or showed cross-hybridisation to multiple loci constituted 16.3 % of the total 1113 potential NAT-detecting probe sets (Fig. 1). A further 26.2 % were classified as "sense" transcript-detecting probe sets, in agreement with the gene symbol annotation provided by NetAffx. However, 28.8 % (320/1113) were classified as "antisense" because they showed indeed a reverse complementary orientation to the sense transcript, confirming the NetAffx annotation of "Negative Strand Matching Probes", and therefore will measure expression of a NAT for the mRNA indicated by the gene symbol. In addition, 320 other probe sets (28.8 %) detect a protein-coding transcript that overlaps with an mRNA from the opposite DNA strand. In these cases (category "overlap"), the probe set measures the expression of an mRNA that can function also as NAT for the SAS partner transcript. Additional file 1 provides the list of probe sets classified as "antisense" or "overlap", annotated with the gene symbol and the gene title.

For comparison, we manually validated the orientation of a subset of 391 potential NAT-detecting probe sets by performing a Blast search using the probe set target sequence as query in Ensembl. Comparing the results of the automatic and manual classification approaches, we found a high concordance in the category "antisense" where 58/391 were called by the automatic versus 63/391 by the manual method (overlap of 51 probe sets). In the category "overlap", automatic classification gave more hits than manual inspection (120 versus 81 of 391 probe sets). Together, the manual and automatic classification called 653 probe sets on the MOE430 2.0 GeneChip as NAT-detecting probe sets, which are listed in Additional file 1 annotated with the gene symbol and the result of manual and automated validation. This annotation may be used to mine existing microarray data sets for expression and regulation of hundreds of NATs. Based on a rate of 57.5 % of confirmed NAT-detecting transcripts for MOE430 2.0 (640/1113 by the automatic classification), the human U113 Plus 2.0 GeneChip that has 3141 probe sets with a NetAffx annotation of "Negative Strand Matching Probes" can be expected to contain in the range of 1800 NAT-detecting probe sets.

While these large numbers of NAT-detecting probe sets should facilitate the quantitative study of expression and regulation of antisense transcripts, it has to be pointed out that only a fraction of all NATs can be detected by mining MOE430 or U133 expression analysis data sets: Because of the design of MOE430 microarrays, NAT-detecting probe sets are restricted to the 3' region of mRNAs and will usually not detect intronic non-coding RNAs. Since the labeling protocol used in most MOE430 data sets deposited in public databases is specific for poly-adenylated transcripts, all polyA- NATs are also not detected by the NAT-detecting probe sets confirmed in our study.

### Confirmation of MOE430 2.0-detected antisense transcripts by strand-specific RT-PCR

Of the manually identified 144 probe sets detecting NATs on the MOE430 2.0 GeneChip, a significant fraction appears to be expressed based on present calls on at least 3 out of 12 arrays of 41 and 83 percent for "antisense" and "overlap" probe sets in a dataset obtained from macrophages stimulated in vitro (unpublished data). We next tested if the expression of NATs as indicated by these probe sets from the MOE430 2.0 GeneChip could be confirmed using strand-specific RT-PCR as an independent method.

**Figure 2:**
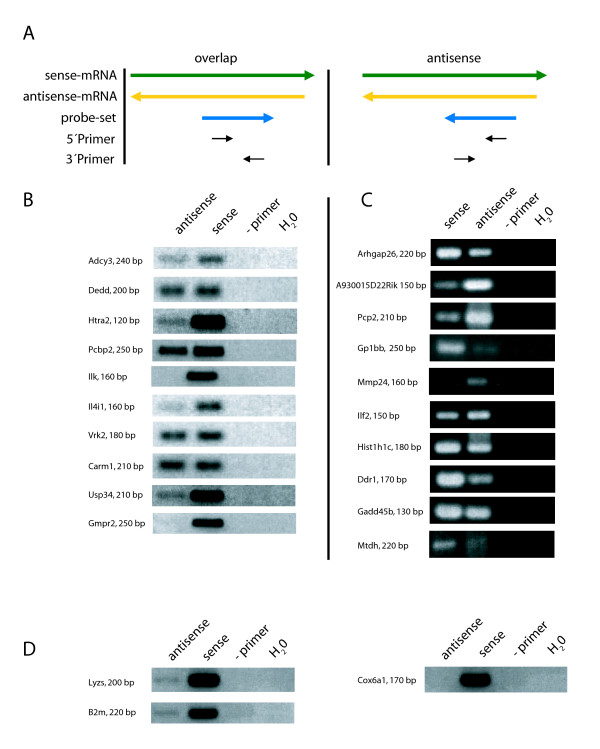
**Confirmation of NAT expression by strand-specific RT-PCR**. A) Schematic illustration of the primer orientation for the strand-specific RT-PCR; left panel: for transcripts of the category "overlap"; right panel: transcripts of the category "antisense". B) RT-PCR products of transcripts of the category "overlap" with indicated product sizes. C) RT-PCR products of transcripts of the category "antisense". D) Strand-specific RT-PCR for three transcripts with no prior evidence of SAS pairing. *Sense*: RT reaction with 3' reverse primer; *antisense*: RT reaction with 5' forward primer; *-primer: *no primer during RT reaction; *H*_2_*O: *no template control.

To this end, we selected ten antisense-detecting probe sets from each of the categories "antisense" and "overlap", for which an additional probe set is present on the MOE430 2.0 GeneChip that detects the corresponding sense transcript and chose primers from the probe set sequences [see Additional file 2]. Probe set- and strand-specific first strand cDNA synthesis was set up using the PCR primers in separate reactions, with the 5' primer generating cDNA of sense transcripts and the 3' primer of antisense transcripts for the category "antisense", whereas the orientation is reversed for probe sets in the "overlap" category (Fig. 2a). Using an RNA pool from different mouse tissues, for all ten probe sets from the "antisense" category and 8/10 form the "overlap" category, antisense transcripts were detected as indicated by the amplification of a PCR fragment of the expected size [see Additional file 2] (Fig. 2b, c). Relative levels of sense and antisense transcripts differed between the probe sets. For comparison, strand-specific RT-PCR for *lyzs*, *b2m *and *cox6a1*, three genes not described to show antisense transcription, was included. While as expected no antisense expression was detectable for *cox6a1*, both *lyzs *and *b2m *PCR showed a band indicating antisense expression. In comparison, the signal for the *lyzs *and *b2m *sense transcripts was much stronger (Fig. 2d), and the fact that the sense RNA-detecting PCR became positive 10 cycles earlier than the antisense-detecting PCR in a SYBRgreen real-time PCR analysis (not shown) confirmed a much more abundant expression. However, the fact that with strand-specific RT-PCR bands suggesting antisense RNA for *lysz *and *b2m *were detected may indicate that using this method absolute strand-specificity cannot be achieved. Therefore, in case of strong expression of the sense RNA, detection of antisense transcripts by strand-specific RT-PCR at low levels may become unreliable. In future work, the use of adapter-tagged primers for RT may prove helpful in circumventing this problem [26]. On the other hand, it should be stressed that this effect cannot account for antisense signals that are within a similar range as the corresponding sense transcripts. Hence, the probe sets we identified as antisense-detecting by manual validation in silico and confirmed as expressed by RT-PCR indeed measure the expression of antisense transcripts.

### Comparison of expression levels of sense-antisense pairs in mouse organs by RT-PCR

**Figure 3:**
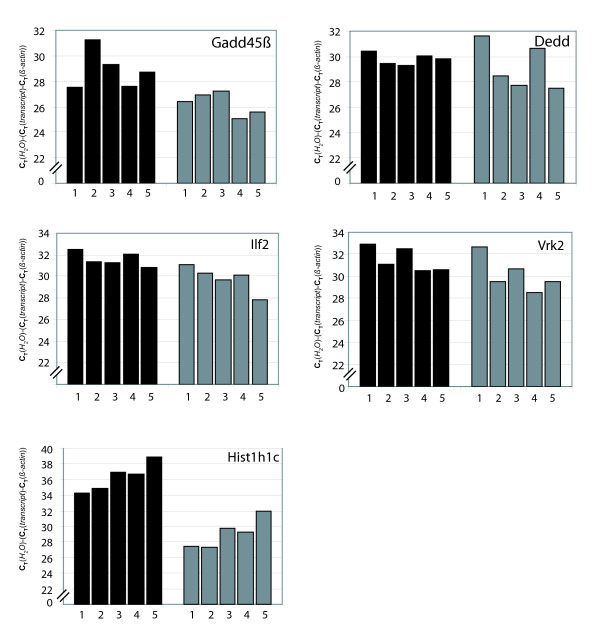
**Quantitative strand-specific RT-PCR analysis of SAS pair expression in different organs**. *1: *liver, *2: *lung, *3: *spleen, *4: *kidney, *5: *thymus. cDNAs were analyzed using SYBR real-time qPCR. Black bars represent sense transcripts, grey bars the corresponding antisense transcripts. SYBR qPCR for β-actin was used for normalization to control for variations in RNA input. The unit is C_T_(H_2_O)-(C_T_(transcript)-C_T_(β-actin)). Data shown are mean values of duplicate determinations.

The results described above obtained using pooled RNA indicated a generally lower expression level of antisense transcripts compared to sense mRNAs. To investigate whether absolute and relative expression levels of antisense transcripts differ between tissues, we analyzed by real-time qPCR the expression of five SAS pairs (*ilf2, vrk2, gadd45b, dedd, hist1h1c*) after strand-specific cDNA synthesis in murine spleen, liver, lung and thymus (Fig. 3). Overall, while expression of antisense transcripts was lower than that of the corresponding sense transcripts, differences in expression levels between tissues appeared to follow the same pattern for sense and antisense transcripts. There were some exceptions to this rule, however, e.g. the high level of Dedd antisense transcript in liver and kidney, and the strong expression of *vrk2 *antisense mRNA in the liver (Fig. 3).

### Correlation of SAS expression in multiple Affymetrix data sets

**Figure 4:**
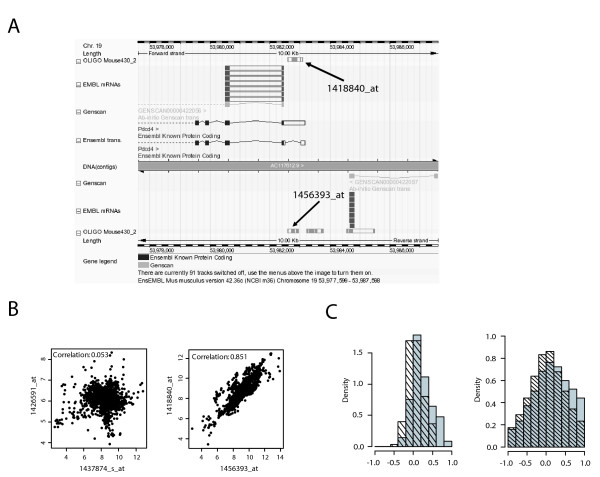
**Correlation analysis of SAS pair expression in multiple MOE430 data sets**. A) Exemplary screenshot of the contig view panel from the ENSEMBL Genome Browser for one of the 179 found probe set pairs with probe sets in sense and antisense orientation, respectively. B) Exemplary scatterplots of the expression values for two SAS probe set pairs. Left: no correlation between probesets 1437874_s_at and 1426591_at; right: positive correlation for the sense (1418840_at) and antisense (1456393_at) probe set pair shown in A). Data represents vsn-normalized and scaled expression values (unit:natural logarithm) from 1637 mouse MOE420 2.0 microarrays downloaded from the Gene Expression Omnibus database. Correlation was calculated using Pearson Correlation. C) *Left panel: *Distribution of Pearson correlation coefficients using the data matrix described in B) for 179 SAS probe set pairs (gray bars) and 150 randomly picked control probeset pairs (hatched bars). *Right panel: *same as left, but with Pearson Correlation performed for each individual dataset.

The presence of a large number of probe sets on the MOE430 2.0 GeneChip that detect antisense transcripts for an mRNA that is probed by a complementary probe set [see Additional file 3] makes it possible to analyze the regulation of SAS transcript pairs by using the large number of Affymetrix microarray data sets deposited in public databases such as Gene Expression Omnibus (GEO) or ArrayExpress. We decided to utilize this wealth of publicly available data to address the question whether SAS transcript pairs are always concordantly regulated, as suggested by the results of our qRT-PCR data (Fig. 3). We downloaded expression data from a total of 1637 MOE430 2.0 GeneChips (derived from 129 different datasets, see Additional file 4 for the GSE numbers) from GEO, scaled the arrays for a median expression of 500 and calculated Pearson's correlation coefficients for 179 SAS and 150 randomly chosen control probe set pairs. Fig. 4a shows an example of a SAS probe set pair depicted in the Ensembl browser. Examples of SAS pairs showing no correlation as well as a high degree of correlation are shown in Fig. 4b. The distribution of correlation coefficients for all SAS pairs in comparison to the control probe set pairs is depicted in Fig. 4c. When correlation was analysed using the pooled data from all experiments (left panel), the correlation coefficients for the control probe set pairs are distributed roughly symmetrically around 0, however, the distribution is shifted to the right for the SAS pairs (p = 1.09*10^-6 ^using Kolmogorov-Smirnov test). In fact, 20 of 179 SAS probe set pairs were correlated positively with a coefficient of correlation > 0.6, while none of the control pairs passed this threshold. Strikingly, there was not a single SAS pair with a coefficient of correlation < -0.4, suggesting that negative correlation of expression between SAS pairs is a rare event (Fig. 4c, left panel). Pooling the data from all 129 datasets may obscure positive or inverse correlations between SAS transcripts under specific biological conditions. We have therefore also calculated the correlations between SAS and random probe set pairs separately for the individual datasets (Fig. 4c right panel). The distribution of the correlation coefficients from this analysis is wider, but again centered around 0. Importantly, SAS pairs showed more positive correlation of expression than random probe set combinations but no evidence of inverse correlation. These data therefore strengthen the concept that the expression of antisense transcripts tends to be positively linked to expression of the sense partner, and do not support a model where antisense transcripts function primarily to down-regulate levels of the sense transcript by transcriptional interference or RNAi-based degradation. Similar conclusions were drawn in the study of Katayama et al., that found an overall correlation between SAS pair expression. On the other hand, the positive correlation of SAS pair expression shown here does not necessarily mean that antisense RNA does not down-regulate sense mRNA levels. Such a function will have to be tested in individual cases by knocking down the antisense transcript and measuring the effect on sense mRNA levels.

## Conclusion

Taken together, we have shown here that Affymetrix MOE430 microarray data sets contain a considerable amount of quantitative information about the expression levels of hundreds of NATs. This information can be uncovered using the NetAffx annotation "Negative Strand Matching Probes" followed by manual or automated in silico validation in Ensembl, as we have done here for the MOE430 2.0 GeneChip, yielding the more than 600 NAT-detecting probe sets provided in Additional file 1. Experimental confirmation by strand-specific RT-PCR showed that most of the NATs indicated by microarray results were indeed expressed, interestingly at different levels between tissues.

Very recently, Werner et al. reported a similar approach to measure NATs by using the first version of the Affymetrix U74 mouse expression analysis GeneChips, that contained a large number of probe sets in reverse complementary orientation [27]. They found a similar present call rate as reported by us for NAT-detecting probe sets on MOE430 arrays and also could confirm the expression of many SAS pairs by RT-PCR. While MOE430 and U133 GeneChips harbor a much lower percentage of "Negative Strand Matching Probes" probe sets than the U74 mouse set, the large and rapidly expanding number of publicly accessible datasets provides the unprecedented opportunity to extract hidden information about SAS transcript pairs expression as a by-product of gene expression profiling experiments.

## Methods

### Validation of the NetAffx probeset annotation "Negative Strand Match Probes" using the Ensembl genome browser

The manual and the automated probe set annotation are based on Ensembl [25] version 36 (released December 2005). Ensembl defines the probe set mapping to the genome and probe set association to genes as follows: each probe on the Affymetrix MOE430 2.0 array is mapped directly to the genome sequence. This mapping is not necessarily unique. Each probe set is then associated with one or more Ensembl genes by directly comparing the probe set to the set of cDNAs created from the Ensembl transcripts. Each transcript is associated with only one gene (although one gene may have many transcripts).

By manual inspection, the binding region was checked for the orientation of Ensembl known transcripts relative to the probe set. As the GeneChip microarray method analyzes cRNA, the orientations were evaluated as follows: If there is an annotated transcript in the same orientation and no annotated transcript in the reverse orientation, the probeset detects only sense transcripts (category "sense"). If there is an annotated transcript in the reverse orientation and no annotated transcript in the same orientation, the probe set detects only antisense transcripts (category "antisense"). If the probeset matches a genomic region where two annotated transcripts overlap, it detects transcripts that are sense and antisense (category "overlap").

In the automated classification, for a probe set to be considered "sense" to a gene, we required that the probe set be associated with only one gene and that the probes from the probe set are mapped to the genome on the same strand and overlapping the start of the gene by at least one base pair. "Antisense" probe sets map to the genome in a region where a gene is annotated on the opposite strand. Additionally, we require that "antisense" probe sets are not associated with any gene and have no mapped probes that overlap a gene on the same strand. Probes were classified as "cross-hybridizing" if they were associated with more than one gene and as "no transcript" if they are not associated with any gene and have no mapping that overlaps a gene by one base pair. Probes are classified as "overlap" if the genes they are associated with overlaps with a gene on the opposite strand by at least one base pair. This overlap is almost always associated with the untranslated regions.

### RNA preparation and strand-specific reverse transcription PCR (RT-PCR)

Mouse organs were homogenized in peqGOLD TriFast (Peqlab) with an rotor-stator homogenizer, followed by extraction of RNA according to the manufacturer's instructions. Strand specific reverse transcription was performed using either the 5' or the 3' primer designed for a specific gene. The reaction was carried out in 10 μl on 100 ng RNA for one hour at 45°C using Superscript II (Invitrogen) and at 55°C using Superscript III. Subsequent PCR was performed with 5 μl of the RT reaction and the complemented primer (5' or 3') for 30 cycles for endpoint agarose gel analysis. Real-time qPCR using SYBRgreen was done for 40 cycles on an Applied Biosystems 7700 SDS.

## Abbreviations

NAT natural antisense transcript

SAS sense-antisense

## Authors' contributions

This work was done as part of the Master's thesis of SO.

SO perfomed in silico validation of probe sets and strand-specific RT-PCR experiments. JM and RL conceived the study, designed experiments and supervised SO. PF developed the automated probe set classification. JM retrieved and analyzed published data sets for correlation of SAS pairs. RL wrote the paper with input from JM (preparation of figures), PF and SO (Methods). All authors read and approved the final version of the manuscript.
